# Tertiary lymphoid structures are associated with higher tumor grade in primary operable breast cancer patients

**DOI:** 10.1186/s12885-015-1116-1

**Published:** 2015-03-06

**Authors:** Stine L Figenschau, Silje Fismen, Kristin A Fenton, Christopher Fenton, Elin S Mortensen

**Affiliations:** 1 RNA and Molecular Pathology Research Group, Department of Medical Biology, Faculty of Health Sciences, University of Tromso, N-9037 Tromso, Norway; 2 Department of Pathology, University Hospital of North Norway, N-9038 Tromso, Norway; 3 The Microarray Platform, Faculty of Health Sciences, University of Tromso, N-9037 Tromso, Norway

**Keywords:** Immune cell infiltration, Tertiary lymphoid structures, Breast cancer, Tumor, Adaptive immune response

## Abstract

**Background:**

Tertiary lymphoid structures (TLS) are highly organized immune cell aggregates that develop at sites of inflammation or infection in non-lymphoid organs. Despite the described role of inflammation in tumor progression, it is still unclear whether the process of lymphoid neogenesis and biological function of ectopic lymphoid tissue in tumors are beneficial or detrimental to tumor growth. In this study we analysed if TLS are found in human breast carcinomas and its association with clinicopathological parameters.

**Methods:**

In a patient group (n = 290) who underwent primary surgery between 2011 and 2012 we assessed the interrelationship between the presence of TLS in breast tumors and clinicopathological factors. Prognostic factors were entered into a binary logistic regression model for identifying independent predictors for intratumoral TLS formation.

**Results:**

There was a positive association between the grade of immune cell infiltration within the tumor and important prognostic parameters such as hormone receptor status, tumor grade and lymph node involvement. The majority of patients with high grade infiltration of immune cells had TLS positive tumors. In addition to the degree of immune cell infiltration, the presence of TLS was associated with organized immune cell aggregates, hormone receptor status and tumor grade. Tumors with histological grade 3 were the strongest predictor for the presence of TLS in a multivariate regression model. The model also predicted that the odds for having intratumoral TLS formation were ten times higher for patients with high grade of inflammation than low grade.

**Conclusions:**

Human breast carcinomas frequently contain TLS and the presence of these structures is associated with aggressive forms of tumors*.* Locally generated immune response with potentially antitumor immunity may control tumorigenesis and metastasis. Thus, defining the role of TLS formation in breast carcinomas may lead to alternative therapeutic approaches targeting the immune system.

## Background

A growing number of publications has described the complex architecture of immune cell infiltration in human solid tumors [1]. Immune responses may develop ectopically at sites of inflammation or infection independently of secondary lymphoid organs [2-4]. The cellular composition of immune cell infiltrates in the tumor microenvironment varies and is heterogeneous, containing innate immune cells such as macrophages, dendritic cells, natural killer cells, granulocytes and mast cells [1]. In addition cells of the adaptive linages, B and T lymphocytes have been observed. Premalignant and *in situ* lesions, as well as invasive carcinomas of the breast, contain immune cell infiltrates in the neoplastic stroma, indicating that the tumor progression is linked to abundant infiltration of immune cells [5]. Several studies support the notion that spontaneous adaptive responses can be elicited by the host against tumor cells. This is believed to be a specific anti-tumor response rather than randomly recruited lymphocytes from the circulation [6-9]. The notable presence of these immune cells, especially the lymphocytes and antigen presenting dendritic cells, has provided evidence that certain tumors can elicit such an immune response. The development of ectopic lymphoid tissue, or tertiary lymphoid structures (TLS), in tumors has been described in several other neoplastic diseases such as lung cancer [10,11], colorectal cancer [12,13], malignant melanoma [14,15], as well as being a key feature of chronic inflammatory autoimmune and infectious diseases, including rheumatoid arthritis [16,17], Sjögren’s syndrome [18,19], and Helicobacter pylori-induced gastritis [20,21].

TLS that develop within the tumor resemble the organization of immune cells in secondary lymphoid organs, in that they contain follicles comprising B lymphocytes and follicular dendritic cells (FDC), with surrounding areas of T lymphocytes and subpopulations of dendritic cells (DC). Following antigen stimulation, B lymphocytes and follicular helper T (Tfh) cells in the B cell zone of these follicles express Bcl6 which is unique for germinal centers (GC) [22]. High endothelial venules (HEV), blood vessels specialized for regulation of lymphocyte trafficking from lymphatic organs into peripheral tissues, are also described in breast tumors [23]. HEV are participating in the development and maintenance of chronic inflammation as they are essential for regulating the extravasation of lymphocytes into the inflamed areas and tumor tissue. HEV are normally not found in non-neoplastic tissue. They are generally restricted to lymphoid tissues and organs, indicating the importance of specialized vascular systems in the development of TLS [23].

The patient’s prognosis and the clinical outcome of breast cancer are influenced by tumor related factors, including histological tumor grade, tumor size, lymph node involvement and hormone receptor status [24]. Several studies describe the relationship between immune contexture in tumors and the impact on patients’ clinical outcome. Tumors with higher numbers of immune cell infiltrates, especially lymphocytes, are in general associated with improved survival [25,26]. Patients with tumor infiltrating T lymphocyte populations are shown to have favourable clinical outcome, especially tumors with higher levels of CD8+ T lymphocytes are associated with better patient survival rates [27-29]. Even though tumor infiltrating CD20+ B lymphocytes play a role in anticancer immune responses and are a common occurrence in breast tumors [9,30], the role in patients’ clinical outcome is still unclear. It is postulated that B lymphocytes are an independent predictor associated with patients’ outcome and associated with higher tumor grade [31,32]. However, the current opinion is based on conflicting results, suggesting that further studies have to determine whether B lymphocytes play a role in tumor progression and in prediction of cancer specific survival [33].

Consistent with previous findings, tumors behave as triggers for inflammation and the complex interaction between tumor cells and the host inflammatory response is a key feature of carcinogenesis [5,34]. Several studies have shown an important relationship between tumor infiltrating immune cells and the clinical outcome for breast cancer patients. However, it is still unclear if a locally produced immune response, with the formation of TLS within the tumor will have an influence on the development of cancer and patients survival. Although the presence of organized immune cell aggregates in primary operable breast cancers has been shown previously [6,8,22,23,35-37], this is, to our knowledge, the first time the characterization of TLS has been described in a larger patient group of breast carcinomas with its association to clinicopathological features. Taken together, our main results showed more organized immune cell aggregates in tumors with higher grade of immune cell infiltration compared with less inflamed tumors. The presence of intratumoral TLS was associated with higher degree of immune cell infiltration and higher histological tumor grade.

## Methods

This study was approved by the Regional Committees for Medical and Health Research Ethics (REC; Norway, 2010/1523). All analyses were performed on tissue specimen previously collected for diagnostic purposes. The study was considered of significant interest for society, the participant’s welfare and integrity was safeguarded and the material was anonymized. Since all these criteria were fulfilled the Regional Ethics committee agreed to use the material for study purposes.

### Clinical samples

This study was conducted on patients who underwent primary surgery between 2011 and 2012 at the University hospital of North Norway (UNN), Tromsø. We used archived formalin-fixed paraffin-embedded (FFPE) specimens obtained from the Department of Pathology (UNN) with the corresponding hematoxylin and eosin (HE) slides from all patients. A total of 290 patients with invasive carcinoma of no special type (NST), formerly known as invasive ductal carcinoma (IDC) [38], invasive lobular carcinoma (ILC), ductal carcinoma in situ (DCIS) and other types of invasive breast carcinomas were included in the study. None of the patients included in this study received adjuvant therapy before surgery, nor did they have any other known malignant diseases. Patient demographics and baseline clinicopathological characteristics are shown in Table 1. DCIS grade was evaluated according to the Van Nuys classification [39]. Histological tumor grade was assessed by the Nottingham Grading System [40]. The cut off values for Estrogen (ER) and progesterone (PR) were 10%. Tumors demonstrating HER2 protein overexpression or amplified HER2 gene (IHC 3+ or FISH HER2 gene ratio ≥2) were considered to be positive.

**Table 1:** **Patients’ demographics and clinicopathological characteristics (n = 290)**

Age at diagnosis	Patients (n, %)
<40	7 (2.4)
40-50	53 (18.3)
51-60	71 (24.5)
>60	159 (54.8)
Diagnosis	
Invasive carcinoma (NST)	208 (71.7)
ILC	31 (10.7)
DCIS	33 (11.4)
Other	18 (6.2)
DCIS status*	
Invasive carcinomas without DCIS	93 (36.3)
Invasive carcinomas with DCIS	163 (63.7)
DCIS grade	
DCIS grade 1-2	77 (39.3)
DCIS grade 3	119 (60.7)
Hormone receptor status	
ER neg / pos / na	44 (15.2) / 210 (72.4) / 36 (12.4)
PR neg / pos / na	59 (20.3) / 122 (42.1) / 109 (37.6)
HER2 neg / pos / na	215 (74.1) / 40 (13.8) / 35 (12.1)
Tumor size	
≤20 mm	159 (62.1)
21- 50 mm	87 (34.0)
>50 mm	10 (3.9)
Histological grade*	
1	85 (33.3)
2	121 (47.5)
3	49 (19.2)
Lymph node involvement	
Negative	204 (70.3)
Positive	86 (29.7)
Involved lymph nodes	
1-3	62 (72.1)
>3	24 (27.9)
Immune cell infiltration*	
No infiltration	39 (13.5)
Mild infiltration	144 (49.8)
Moderate infiltration	90 (31.1)
Extensive infiltration	16 (5.6)
Aggregate formation*	
Negative	143 (49.5)
Positive	146 (50.5)
TLS formation*	
Negative	175 (61.4)
Positive	110 (38.6)

*Abbreviations: NST* invasive carcinoma of no special type, *ILC* invasive lobular carcinoma, *DCIS* ductal carcinoma in situ, other invasive carcinomas include: tubular carcinoma, cribriform carcinoma, mucinous carcinoma, medullary carcinoma, apocrine carcinoma, metaplastic carcinoma, papillary carcinoma, *ER* estrogen receptor, *PR* progesterone receptor, *HER2* human epidermal growth factor receptor 2, *TLS* tertiary lymphoid structures, *na* not analysed. *Patient(s) data missing.

### Assessment of tumor immune cell infiltrate

Histopathological analysis of full-faced HE stained tissue sections were used to assess the overall level of immune cell infiltration in the breast tumors. Using routine histology, the patient samples were evaluated based on the total amount of immune cell infiltrate, both in the central areas of the tumor and at the invasive margin, then categorized into the following groups: no immune cell infiltrate, mild infiltrate, moderate infiltrate and extensive immune cell infiltrate. By applying this definition, we further divided the categories into two groups: low grade and high grade infiltration of immune cells. The two pathologists (ESM and SF) independently performed the categorizing and had no knowledge of the patients’ background history.

### Immunohistochemistry

Tumor samples that contained organized aggregates of immune cells, judged by HE staining, were further assessed by immunohistochemical analyses. FFPE serial sections (4 μm) were deparaffinized and dehydrated in xylene and graded alcohols. Antigen retrieval was performed by microwave treatment in 10 mM sodium citrate buffer (pH 6.0) for 20 min. Endogenous peroxidase activity was blocked with 3% H_2_O_2_ for 10 min and non-specific binding was blocked with 10% goat serum (Invitrogen™, Life Technologies) for 30 min. Sections were incubated with unlabelled primary antibody for 30 min and Polink-2 HRP Plus DAB kit (Golden Bridge International, Inc., USA) was used as detection system according to the manufacturers’ protocol. For the detection of PNAd + HEV, sections were incubated with purified goat anti-rat light chain specific HRP conjugated polyclonal antibody (1:500, AP202P; Millipore) for 30 min prior to reaction with DAB substrate-chromogen (Golden Bridge International, Inc., USA). Finally, sections were counterstained with hematoxylin and rehydrated in graded alcohols and xylene. All reactions were performed at room temperature, if not stated otherwise. Human lymph node, tonsil and breast tumor were used as positive controls. Negative controls were performed by omitting the primary antibody. Immunohistochemical analyses using the platform specific assays on BenchMark XT (Ventana Medical systems Inc., USA) were performed according to the manufacturers’ instructions. Antibodies used for immunohistochemical analyses are summarized in Table 2.

**Table 2:** **Primary antibodies used for immunohistochemical analyses**

Antigen	Manufacturer	Cat. no	Clone	Species	Control	Dilution
Bcl6	Ventana	760-4241	GI191E/A8	Mouse IgG_1_	Lymph node	Pre-diluted
CD3	Ventana	790-4341	2GV6	Rabbit IgG	Lymph node	Pre-diluted
CD4	Ventana	790-4423	SP35	Rabbit	Lymph node	Pre-diluted
CD8	Ventana	790-4460	SP57	Rabbit	Lymph node	Pre-diluted
CD20	Ventana	760-2531	L26	Mouse IgG_2a_/κ	Lymph node	Pre-diluted
CD21	Ventana	760-4245	2G9	Mouse IgG_2a_	Lymph node	Pre-diluted
CD21	Ventana	760-4438	EP3093	Rabbit IgG_1_	Lymph node	Pre-diluted
CD21	Abcam	ab75985	EP3093	Rabbit IgG	Tonsil	1:100
ER	Ventana	790-4325	SP1	Rabbit IgG	Breast carcinoma	Pre-diluted
PR	Ventana	790-4296	1E2	Rabbit IgG	Breast carcinoma	Pre-diluted
HER2/*neu*	Ventana	790-2991	4B5	Rabbit	Breast carcinoma	Pre-diluted
PNAd	Biolegend	120801	MECA-79	IgM,κ	Lymph node	1:50

### Statistical analyses

Determination of interobserver agreement was assessed by the Cohen’s kappa statistics (κ). Values of κ from 0.60 to 0.79 are considered good, and above 0.80 excellent. Baseline descriptive statistics are reported as frequencies and percentages. Interrelationship between variables was assessed using contingency tables; Phi analyses for dichotomous variables and Spearman rank order correlation for ranked data. The variables with the highest level of significance and important prognostic factors were entered into a regression model. Binary logistic regression analysis with the fixed entry method was performed in order to identify significant predictors for the presence of TLS in patient tumors. The following diagnostic predictors were included in the regression analysis together with the lymphocytic parameters: tumor size, histology grade and clinical nodal status. For all statistical analyses, if not stated otherwise, p values < 0.05 (two-tailed) were considered statistically significant. Statistical analyses were performed using SPSS software version 22 (SPSS Inc., Chicago, IL, USA).

## Results

The demographics and baseline clinicopathological characteristics of patients with primary operable breast carcinomas included are shown in Table 1.

### Characterization of tertiary lymphoid structures in breast carcinomas

**Figure 1:**
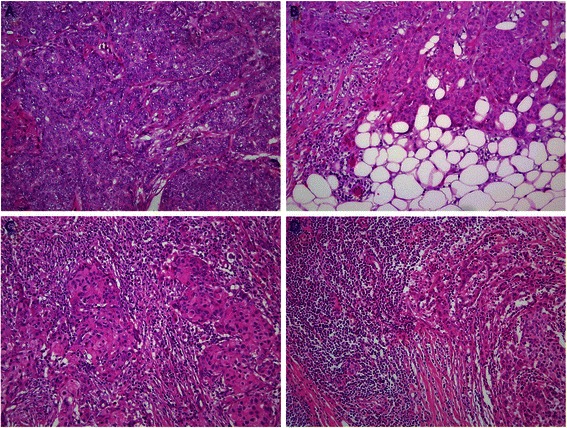
**Characterization of immune cell infiltrate in breast carcinomas.** Invasive human breast carcinomas with **A)** no immune cell infiltrate **B)** mild infiltrate **C)** moderate infiltrate and **D)** extensive infiltrate. All slides are HE stained and at the same magnification (100X).

Tumors from all patients who underwent primary surgery in the period 2011 to 2012 were categorized into four groups based on the degree of immune cell infiltration as shown in Figure 1A-D. The distribution of patient samples showed that 13.5% had no immune cell infiltration (Figure 1A), 49.8% had mild infiltration (Figure 1B), 31.1% had moderate infiltration (Figure 1C), and 5.6% were categorized as tumors with extensive infiltration of immune cells (Figure 1D and Table 1). The interobserver κ value for the categorical parameter (no infiltrate, mild infiltrate, moderate infiltrate, extensive infiltrate) was 0.78 (*p* <0.001).

**Figure 2:**
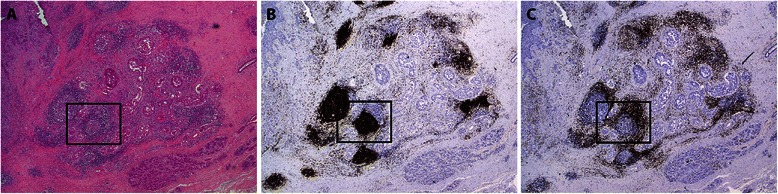
**Representative overview of lymphocytic infiltrate in breast tumor.** Histology and immunohistochemical analyses performed on breast tumor biopsies show the localization and distribution of lymphocytes in primary tumor of the breast. **A)** HE staining shows intratumoral TLS with GC, **B)** CD20+ B lymphocytes forming follicles with surrounding area of **C)** CD3+ T lymphocytes, resembling highly organized structures of secondary lymphoid tissue. Magnification 20X. Higher magnification of boxed area is shown in Figure 3.

**Figure 3:**
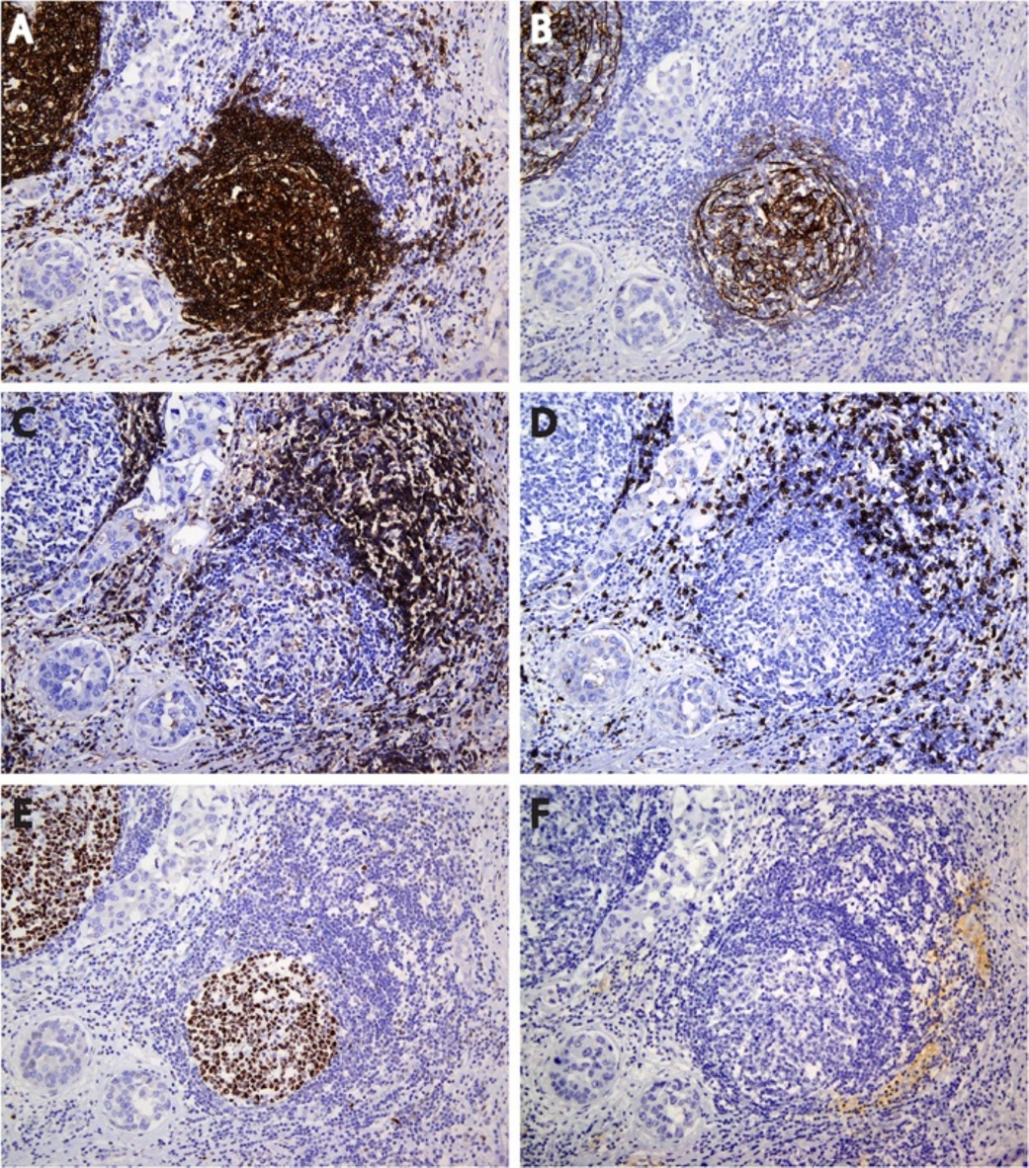
**Characterization of tertiary lymphoid structures in breast carcinoma.** Immunohistochemical detection of the indicated antigens in serial sections of breast carcinoma with extensive immune cell infiltration. Tertiary lymphoid structures with germinal center formation were detected by **A)** CD20+ B lymphocyte follicle comprising a network of **B)** CD21+ FDC, with **C)** CD4+ and **D)** CD8+ T lymphocytes in the T cell zone. **E)** Bcl6+ germinal center B lymphocytes and Tfh cells were also observed within the B cell follicle with surrounding **F)** PNAd + HEV like vessels. Positively stained antigens are shown by brown DAB staining. Magnification 100X.

The organization of tumor infiltrating immune cells was characterized by immunohistochemical detection (Figures 2 and 3). The immune cell aggregates which were TLS positive showed the presence of CD20+ B lymphocytes within the follicles, with areas of CD3+ T lymphocytes resembling the highly organized structures of secondary lymphoid organs (Figure 2B and C). CD21+ FDC formed a tight network in the B cell zone within the follicle as shown in Figure 3A and B. Consistent with previous findings [9], the majority of CD3+ T lymphocytes in the T cell zone were of the CD4+ T cell subset (Figure 3C). CD8+ T lymphocytes were moderately dispersed within the T cell zone (Figure 3D). Moreover, Figure 3E shows Bcl6+ GC B lymphocytes and Tfh cells which were detected in most of the organized lymphoid aggregates. HEV, although restricted to lymphoid tissue, were also found in the tumor tissue adjacent to the aggregates. HEV were typically found in the T cell area of the lymphoid aggregates as shown in Figure 3F. These vessels were not found at other sites than within the TLS, nor in non-cancerous areas of the breast (results not shown). Histopathological analyses revealed immune cell infiltrates with aggregate formation in 50.5% of the patient samples of which 38.6% were TLS positive (Table 1). Intratumoral TLS formation was found both in the periphery of the tumor, the centre of the tumor as well as close to adjacent tumor nests. The extent and size of these structures within the tumor varied in the patient samples.

### Association between immune cell infiltration and clinicopathological parameters

The relationship between the degree of immune cell infiltration in tumors and patients’ clinicopathological characteristics are shown in Table 3. The majority of the patients who had tumor with high grade immune cell infiltration (n = 106) were over 50 years, had invasive carcinoma (NST) and accompanying DCIS component within the tumor. None of these variables were significantly associated with higher grade of immune cell infiltration. ER and PR hormone receptor status were negatively associated (rϕ = −0.394 and −0.342, respectively, p < 0.01) with the grade of immune cell infiltration compared with HER2 status which was positively associated (rϕ = 0.294, p < 0.01). There was a positive association between the total level of immune cell infiltration in tumors and tumor grade (r_s_ = 0.384, p < 0.01). Patients who had higher grade of immune cell infiltration also had higher tumor grades, where 47.2% of the patients had tumor grade 2 and close to 40% had tumor grade 3. There was a weak positive association between the level of immune cell infiltrate and lymph node invasion (rϕ = 0.180, p < 0.01).

**Table 3:** **Association between immune cell infiltration grade in tumors and clinicopathological parameters**

	Low grade immune cell infiltrate (n = 183)	High grade immune cell infiltrate (n = 106)	r value	*p*value
Age (≤50 / >50 years)	36 (19.7) / 147 (80.3)	24 (22.6) / 82 (77.4)	rφ = −0.035	0.549
Invasive carcinoma (NST) / ILC / DCIS / Other invasive carcinomas	130 (71.0) / 22 (12.0) / 16 (8.7) / 15 (8.2)	77 (72.6) / 9 (8.5) / 17 (16.0) / 3 (2.8)	r_s_ = −0.019	0.743
DCIS status (Invasive DCIS − / Invasive DCIS +)	61 (36.7) / 105 (63.3)	31 (34.8) / 58 (65.2)	rφ = 0.019	0.761
DCIS grade (1–2 / 3)	62 (51.2) / 59 (48.8)	15 (20.0) / 60 (80.0)	rφ = 0.311	<0.01
Estrogen receptor status (ER − / ER +)	10 (6.1) / 154 (93.9)	33 (37.1) / 56 (62.9)	rφ = −0.394	<0.01
Progesterone receptor status (PR − / PR +)	20 (18.9) / 86 (81.1)	38 (51.4) / 36 (48.6)	rφ = −0.342	<0.01
HER2 status (HER2 − / HER2 +)	152 (92.1) / 13 (7.9)	62 (69.7) / 27 (30.3)	rφ = 0.294	<0.01
Tumor size (≤20 / 21–50 / >50 mm)	111 (66.9) / 47 (28.3) / 8 (4.8)	47 (52.8) / 40 (44.9) / 2 (2.2)	r_s_ = 0.122	0.052
Tumor grade (I / II / III)	72 (43.6) / 78 (47.3) / 15 (9.1)	13 (14.6) / 42 (47.2) / 34 (38.2)	r_s_ = 0.384	<0.01
Lymph node (− / +)	140 (76.5) / 43 (23.5)	63 (59.4) / 43 (40.6)	rφ = 0.180	<0.01
Involved lymph node (0 / 1–3 / >3)	140 (76.5) / 33 (18.0) / 10 (5.5)	63 (59.4) / 29 (27.4) / 14 (13.2)	r_s_ = 0.187	<0.01

*Abbreviations: NST* invasive carcinoma of no special type, *ILC* invasive lobular carcinoma, *DCIS* ductal carcinoma in situ, *ER* estrogen receptor, *PR* progesterone receptor, *HER2* human epidermal growth factor receptor 2, *r*_*s*_ spearman rank order correlation, *r*φ phi coefficient. A small number of patients did not have complete data. Data presented as n (%).

### Association between TLS formation and clinicopathological parameters

The association between the presence of TLS in tumors and patients’ clinicopathological characteristics are shown in Table 4. There was a strong positive association (rϕ = 0.796, p < 0.01) between detection of TLS and immune cell aggregates formed in tumors. Tumor grade and the level of immune cell infiltration were weakly associated with TLS formation (rϕ = 0.294 and 0.567, respectively, p < 0.01). We observed a negative association between TLS formation and ER and PR positive tumors as more TLS negative tumors were ER and PR positive (rϕ = −0.342 and −0.308, respectively, p < 0.01). A weak association between TLS positive tumors and the presence of HER2 receptor was observed within the tumors studied, although more TLS negative tumors were HER2 negative (rϕ = 0.243, p < 0.01).

**Table 4:** **Association between presence of tertiary lymphoid structures in tumors and clinicopathological parameters**

	TLS negative tumors (n = 175)	TLS positive tumors (n = 110)	r value	*p*value
Age (≤50 / >50 years)	38 (21.7) / 137 (78.3)	21 (19.1) / 89 (80.9)	rφ = 0.032	0.595
Invasive carcinoma (NST) / ILC / DCIS / Other invasive carcinomas	122 (69.7) / 22 (12.6) / 18 (10.3) / 13 (7.4)	81 (73.6) / 9 (8.2) / 15 (13.6) / 5 (4.5)	r_s_ = −0.038	0.518
DCIS status (Invasive DCIS − / Invasive DCIS +)	59 (37.8) / 97 (62.2)	30 (31.6) / 65 (68.4)	rφ = 0.063	0.316
DCIS grade (1–2 / 3)	52 (45.2) / 63 (54.8)	24 (30.0) / 56 (70.0)	rφ = 0.153	<0.05
Estrogen receptor status (ER − / ER +)	10 (6.5) / 144 (93.5)	31 (32.6) / 64 (67.4)	rφ = −0.342	<0.01
Progesterone receptor status (PR − / PR +)	20 (19.6) / 82 (80.4)	36 (48.6) / 38 (51.4)	rφ = −0.308	<0.01
HER2 status (HER2 − / HER2 +)	141 (91.0) / 14 (9.0)	69 (72.6) / 26 (27.4)	rφ = 0.243	<0.01
Tumor size (≤20 / 21–50 / >50 mm)	99 (63.5) / 48 (30.8) / 9 (5.8)	56 (58.9) / 38 (40.0) / 1 (1.1)	r_s_ = 0.025	0.694
Tumor grade (I / II / III)	64 (41.3) / 75 (48.4) / 16 (10.3)	19 (20.0) / 45 (47.4) / 31 (32.6)	r_s_ = 0.294	<0.01
Lymph node (− / +)	130 (74.3) / 45 (25.7)	70 (63.6) / 40 (36.4)	rφ = 0.113	0.056
Involved lymph node (0 / 1–3 / >3)	130 (74.3) / 34 (19.4) / 11 (6.3)	70 (63.6) / 27 (24.5) / 13 (11.8)	r_s_ = 0.120	<0.05
Immune cell infiltration (low / high)	149 (85.1) / 26 (14.9)	32 (29.1) / 78 (70.9)	rφ = 0.567	<0.01
Aggregate formation (− / +)	143 (81.7) / 32 (18.3)	0 / 110 (100)	rφ = 0.796	<0.01

*Abbreviations: TLS* tertiary lymphoid structures, *NST* invasive carcinoma of no special type, *ILC* invasive lobular carcinoma, *DCIS* ductal carcinoma in situ, *ER* estrogen receptor, *PR* progesterone receptor, *HER2* human epidermal growth factor receptor 2, *r*_*s*_ spearman rank order correlation, *r*φ phi coefficient. A small number of patients did not have complete data. Data presented as n (%).

In order to identify significant predictors for intratumoral TLS formation a regression analysis was conducted using a fixed entry model. The variables of interest that were entered into the binary logistic regression model are shown in Table 5. Histological tumor grade 2 and 3 were associated with TLS formation in univariate analysis. Accordingly, multivariate regression analysis identified tumor grade 3 as independent predictor for TLS formation. As shown in Table 5, the odds ratio of 2.78 [CI, 1.06-7.27] indicates that the odds for having TLS positive tumors were almost three times more likely for grade 3 tumors than for patients with grade 1 tumors. Another significant independent parameter was the degree of immune cell infiltration. The model predicts that the odds of having TLS formation in tumors were more than 10 times higher for patients with higher grade of inflammation than lower graded infiltrated tumors. Hence, patients with high grade inflammation are two times more likely to have TLS formation than not. Tumor size or whether patients had lymph node metastasis did not affect the detection of intratumoral TLS formation.

**Table 5:** **Logistic regression models for predicting TLS formation in breast carcinomas**

	Univariate analysis			Multivariate analysis		
	OR	95% CI	*p*value	OR	95% CI	*p*value
Tumor size (≤20 / 21–50 / >50 mm)						
≤20 mm	1.00	(Reference)		1.00	(Reference)	
21 - 50 mm	1.40	0.82 - 2.40	0.22	0.84	0.42 - 1.70	0.63
>50 mm	0.20	0.02 - 1.59	0.13	0.14	0.01 - 1.42	0.09
Tumor grade (I / II / III)						
Grade 1	1.00	(Reference)		1.00	(Reference)	
Grade 2	2.02	1.08 - 3.80	<0.05	1.53	0.72 - 3.26)	0.27
Grade 3	6.53	2.96 - 14.40	<0.01	2.78	1.06 - 7.27	<0.05
Immune cell infiltration (low / high)	13.97	7.78 - 25.09	<0.01	10.80	5.54 - 21.05	<0.01
Lymph node (− / +)	1.65	0.99 - 2.76	0.057	0.98	0.47 - 2.02	0.95

*Abbreviations: OR* odds ratio, *CI* confidence interval.

## Discussion

It is well established that tumors of a variety of cancer types are commonly infiltrated with immune cells which are organized in structures resembling conventional secondary lymphoid organs [41]. For the first time, we describe intratumoral TLS formation in a larger group of breast carcinoma patients. Our study demonstrates that TLS are frequently found in breast tumors with higher degree of immune cell infiltration and higher histological tumor grade. Breast carcinoma cells are often closely associated with tumor infiltrating lymphocytes [42]. Invasive carcinomas (NST) are the most common type of breast cancer, but unlike subtypes such as medullary and basal-like carcinomas characterized by prominent inflammation, they have a more variable lymphocytic infiltration [43]. Breast carcinomas often contain infiltrating B and T lymphocytes, with dense infiltrates occurring in approximately 20% of tumors, and moderate infiltrates in about 50% [9,43]*.*

Our results showed the presence of tumor-associated TLS in about one third of the breast carcinomas. These structures comprise distinct T cell zones and B lymphocytes segregated into follicles hosting functionally active GC, exhibiting structural analogies with secondary lymphoid tissues. The observed TLS were confined to peritumoral areas, and were detected both in the periphery and central tumor nests*.* We did not observe these structures in non-tumor areas, indicating that these structures were tumor-associated and may be a response to the tumor microenvironment. Consistent with our findings, lymphoid neogenesis has been reported in several other neoplastic diseases [10-15]*.* TLS are also frequently observed in chronic inflammatory conditions in which sustained lymphocyte activation occurs in the presence of persistent antigenic stimuli [16-19]. Whether TLS can generate an intratumoral immune response in a way similar to secondary lymphoid organs is still unclear. Experimental data from mouse models have provided evidence that adaptive immunity can be initiated independently of secondary lymphoid organs [44]. Notably, studies have demonstrated that development of TLS play a role in the induction of a local antitumor immune response in neoplastic tissue in mice lacking peripheral lymph nodes [45-47]. Recent data published by Goc *et al.* suggests TLS as an important site for *in situ* activation of tumor-associated lymphocytes and supports the contribution of these structures in generation of a protective immune response in lung cancer [48]. Furthermore, Gu-Trantien *et al.* demonstrated that CXCL13-producing Tfh cells located in GC of breast tumors are associated with organized lymphoid structures that may produce an antitumor immune reaction [22]. In line with previous works, we observed Bcl6+ B lymphocytes and Tfh cells within GC which argue for functional ectopic centers in breast tumors. Given that extra-nodal activation of lymphocytes occurs in tumor-associated TLS and facilitates induction of local immune reactions, it makes sense that this phenomenon could be beneficial in antitumor immunity.

It has become generally accepted that the immune system exerts a dual role in carcinogenesis. Thus, one can not rule out the opposite protumoral effect as the tumor eradication by the immune system is often inefficient, and spontaneous or complete regression of established tumors are extremely rare [49]. The immunoediting theory emphasizes that during tumor development and progression, there is a dynamic interaction between the host immune system and the developing tumor, a process which describes the host-protective and tumor promoting roles of the immune system [50]. Studies in mouse models suggest that CCL21 secreting tumors may alter the locally generated immune response by promoting tumor-induced tolerance, which facilitates tumor progression [51]. In addition, impairment of antitumoral T cell responses and modulation of immune responses by immune complexes might represent underlying mechanisms that also promote tumor progression [52]. The immunoediting theory was recently further challenged by Ciampricotti *et al.* who evaluated the role of adaptive immune responses in tumorigenesis by establishing a mouse model of spontaneous HER2+ breast tumors. The study demonstrated that the development of tumors were not suppressed by immunosurveillance mechanisms or influenced by adaptive immune responses [53]*.* Interestingly, a recent study showed decreased density of HEV and DC-LAMP+ DCs around the DCIS component compared to invasive areas in breast tumors, suggesting to be a key feature in the progression from *in situ* to invasive carcinoma [37]. However, our results did not show a strong association between the presence of TLS and DCIS components within the invasive tumors. Taken together, there are examples where TLS formation in neoplastic diseases is associated with promotion of tumorigenesis rather than generating a protective immune response. Still, the overall morphology of a conventional secondary lymphoid organ with Bcl6+ GC cells combined with specialized population of DC and distinct T cell area are convincing findings of local adaptive immune response taking place in these structures.

The major finding of our study demonstrated that higher tumor grade was associated with intratumoral TLS formation. Tumors with histological grade 3 are the most aggressive types and are associated with worst prognosis. Lymph node metastases are also associated with worse prognosis independent of tumor grade. Our results demonstrated a weak association between the presence of intratumoral TLS and lymph node involvement. It is therefore important to address whether TLS would influence the clinical outcome in a larger series of breast cancer patients. The formation of TLS with morphologically and immunophenotypically identical features to a conventional secondary lymphoid organ is intriguing, and adds to the findings of similar structures in malignant tumors in other organ systems [10-15]. In addition, as previously mentioned, immune cell infiltration in tumors is associated with prognosis and survival of breast cancer patients. The distinct combination of tumor infiltrating immune cells combined with lymphoid neogenesis may suggest TLS as a biomarker in cancer. Hence, the importance of defining the immunophenotype, the location and the functionality of the immune infiltrates in breast carcinomas may become useful in predicting a patient’s prognosis. Careful studies on the mechanisms of the immune reactions and their impact at different stages of disease should hopefully result in an improved approach to targeted therapies.

## Conclusion

In this study, we characterized tertiary lymphoid structures in breast cancer patients and addressed the question whether there was a relationship between immune cells infiltrating human breast tumors, intratumoral formation of TLS and clinicopathological parameters. Our main findings conclude that tumors with higher degree of immune cell infiltration also have higher tumor grade. In addition, intratumoral TLS formation was associated with higher inflammation grade and higher tumor grade. These findings support the notion that infiltrating immune cells are a common feature in breast cancer tumors and that breast tumors frequently contain tertiary lymphoid structures.

## Abbreviations

Bcl6: B-cell lymphoma 6 protein
CI: Confidence interval
CD: Cluster of differentiation
DAB: 3,3’-Diaminobenzidine
DC: Dendritic cells
DCIS: Ductal carcinoma in situ
ER: Estrogen receptor
FDC: Follicular dendritic cells
FFPE: Formalin-fixed paraffin-embedded
GC: Germinal center
HE: Hematoxylin and eosin
HER2: Human epidermal growth factor receptor 2
HEV: High endothelial venules
HRP: Horseradish peroxidase
IDC: Invasive ductal carcinoma
ILC: Invasive lobular carcinoma
NST: Invasive carcinoma of no special type
OR: Odds ratio
PNAd: Peripheral node addressin
PR: Progesterone receptor
Tfh: Follicular helper T cells
TLS: Tertiary lymphoid structures
